# Supplementary material for: Protocol on a systematic review of nomenclature and outcomes in children with complex critical illness in Paediatric Critical Care: The basis for consensus definition
Source: PLoS One. 2025 Feb 6;20(2):e0318312. doi: 10.1371/journal.pone.0318312 (PMC11801698; doi:10.1371/journal.pone.0318312)
Supplement: S1 Appendix — (DOCX) [file pone.0318312.s002.docx]

# [S1 Appendix.](https://journals.plos.org/plosone/article/file?type=supplementary&id=10.1371/journal.pone.0307470.s001)Search strategy for MEDLINE.

1. exp Critical Care/
2. Critical* Care.mp.
3. critical* ill*.mp.
4. ICU.mp.
5. intensive care units/
6. exp Intensive Care Units, Pediatric/
7. PICU.mp.    7417
8. PCCU.mp.    49
9. ((p?ediatric or child*) and (intensive or ICU)).mp.
10. 1 or 2 or 3 or 4 or 5 or 6 or 7 or 8 or 9
11. exp adolescent/ or exp child/ or exp infan*/
12. (child* or teen* or infan* or adolescen* or p?ediatric or young or youth or Juvenile).mp.
13. 11 or 12
14. exp Chronic Disease/
15. chronic disease*.mp.
16. (complex adj3 need*).mp.
17. (complex adj2 condition*).mp.
18. (complex adj3 condition*).mp.
19. Life-limit*.mp.
20. Lifelimit*.mp.
21. (chronic adj2 illness*).mp.
22. Chronic health.mp.
23. medical complexity.mp.
24. patient complexity.mp.
25. (multi adj3 morbid*).mp
26. Long-term illness.mp.
27. Technology-Dependent Child*.mp.
28. medically fragile.mp.
29. (severe adj2 condition*).mp.
30. neurodisability.mp.
31. prolonged-stay.mp.
32. 14 or 15 or 16 or 17 or 18 or 19 or 20 or 21 or 22 or 23 or 24 or 25 or 26 or 27 or 28 or 29 or 30 or 31
33. 10 and 13 and 32
34. limit 33 to yr="2014 -Current"
