# Supplementary material for: Protocol on a systematic review of nomenclature and outcomes in children with complex critical illness in Paediatric Critical Care: The basis for consensus definition
Source: PLoS One. 2025 Feb 6;20(2):e0318312. doi: 10.1371/journal.pone.0318312 (PMC11801698; doi:10.1371/journal.pone.0318312)
Supplement: S1 Table — (DOCX) [file pone.0318312.s003.docx]

**S1 Table Data extraction form.**

| **Source** | **Eligibility** | **Study Characteristics** | **Methods** | **Patient Demographics** | **Population definition & Key findings or outcome of interest** | **Miscellaneous** |
| --- | --- | --- | --- | --- | --- | --- |
| Study ID (created by review author). | Confirm eligibility for review. | Author name | Study design. | Total number and groups if applicable | How is the study population defined? | Funding source. |
| Review author ID (created by review author). | Reason for exclusion | Title | Total study duration. | Reason for PICU admission | Definition of paediatric complex critical illness | Key conclusions of the study authors. |
| Citation and contact details. |  | Country of origin | Sequence generation. | Diagnostic criteria. | Definition of prolonged PICU admission | Miscellaneous comments from the study authors. |
|  |  | Journal and year of publication | Allocation sequence concealment. | Age. | Definition of medical complexity in PICU | References to other relevant studies. |
|  |  | Clinical setting/type of PICU | Blinding. | Sex. | How the definition was developed and/or validated by primary study. | Correspondence required. |
|  |  |  | Other concerns about bias*. | Functional status (using validated tools such as functional status score) | *For each outcome of interest*:  Outcome definition (with diagnostic criteria if relevant). | Miscellaneous comments by the review authors. |
|  |  |  |  | Severity of illness (using validated tools) | Unit of measurement (if relevant). |  |
|  |  |  |  | Co-morbidities |  |  |
