# Supplementary material for: Protocol on a systematic review of nomenclature and outcomes in children with complex critical illness in Paediatric Critical Care: The basis for consensus definition
Source: PLoS One. 2025 Feb 6;20(2):e0318312. doi: 10.1371/journal.pone.0318312 (PMC11801698; doi:10.1371/journal.pone.0318312)
Supplement: Attachment — Submitted filename: PLOS ONE Reviewer Response 7.11.24.pdf [file pone.0318312.s004.pdf]

Thank you for the opportunity to review this manuscript, which details a systematic review protocol on nomenclature and outcomes in children with complex critical illness in the pediatric ICU.

While this topic is of great importance considering the increasing medical complexity of children admitted to the pediatric ICU, the manuscript is not well organized. For a better organizational structure, I suggest combining PLOS One requirements for main sections (Introduction, Materials & Methods, Discussion) with the PRISMA-P criteria as subsections within each main section. The use of future tense throughout the manuscript is also at times confusing, because it seems that the search strategy has already been formed (Appendix 1) and the trial has already been registered, but these types of activities are stated to occur in the future. Together these two issues make it difficult to clearly understand the review methods.

I have the following additional specific suggestions to improve clarity and quality for publication:

<u>Abstract:</u>

- There should not be references within an abstract.
- The abstract and main text should follow the same headings.
- Only include abbreviations that are used again in the abstract.

<u>Introduction</u>

- The review purpose/objectives are stated multiple times in multiple different ways. One concise objective or set of aims is needed.
- Additionally, the review objective(s) seems quite broad. Understanding how pediatric chronic critical illness (PCCI) is defined, who the children with PCCI are, and the outcomes they are experiencing feels like 3 separate literature reviews, and will be difficult to complete systematically using one search strategy, data extraction tool, etc.
    - If outcomes remain a focus of the review, the core outcomes set developed for pediatric critical care by Fink et al., 2020 should be mentioned. How is your outcomes aim different from this work?
      Fink EL, Jarvis JM, Maddux AB, et al. Development of a core outcome set for pediatric critical care outcomes research. *Contemporary Clinical Trials*. 2020;91:105968. doi:10.1016/j.cct.2020.105968
- A clearer reason is needed for how this review is different from the Zorko et al., 2023 scoping review.
- Edwards et al., 2022 review also needs mentioned earlier, and how it is distinct from the review you are undertaking.
- I do not believe Table 1 is needed, and in fact is confusing as the authors have already stated that there are not commonly accepted definitions for these concepts. I think it would be adequate to state that chosen search terms were based on concepts identified in the Edwards et al., 2022 review.
- Things being mentioned in the Introduction belong in the Methods (e.g., eligibility criteria,

types of sources).
  - Article keywords do not belong in the main text.
  - What is the COMET theoretical framework? It is mentioned without reference or explanation. Is this where the following Concept and Context sections come from, because I also found those a bit confusing and redundant from what had already been described.
- Again, exploring all types of literature with such a broad research question feels quite difficult. Is there a specific reason grey literature needs to be included? Won't the inclusion of systematic reviews be redundant given the inclusion of original research articles? Perhaps the reference list of reviews can be searched for relevant citations but don't need to be included themselves?

<u>Methods</u>
- Authors should state that they did (not "will") follow PRISMA-P when reporting protocol and reference Appendix 3 at beginning of methods section. Use PRISMA-P criteria to organize manuscript.
- Use of multiple article reviewers and close consultation with librarian are strengths of the review methods.
- Authors state that they "will" register protocol on PROSPERO, but it seems that they already have? Please reference this in Abstract and Methods.
- Appendices are incorrectly numbered/ identified in text.
- A pilot test is mentioned in Study/Source of Evidence selection section, what does this mean?
- CASP abbreviation should be defined and referenced in Quality Assessment section. A description of the tool is needed.
- Patient and public involvement section feels vague, is there a specific parent advisory group that was consulted on methods?
- Dissemination strategies are mentioned multiple times and feel redundant (speaks to need for better manuscript organization). Authors state they "will" publish protocol in academic pediatric critical care literature which feels confusing to read in a protocol paper. Also, while PLOS ONE is a great journal, I would not call it a pediatric critical care journal?
- Multiple ethics sections – again feels redundant.

<u>Discussion/Conclusion</u>
- Discussion section should be a discussion of the review protocol, not a report of what will be discussed in the results manuscript. Should include strengths/ limitations of review methods, how it builds on previous reviews, and how this review will be used to build the consensus definitions (which is currently included in the Methods and should be moved).
