# Supplementary material for: Protocol on a systematic review of nomenclature and outcomes in children with complex critical illness in Paediatric Critical Care: The basis for consensus definition
Source: PLoS One. 2025 Feb 6;20(2):e0318312. doi: 10.1371/journal.pone.0318312 (PMC11801698; doi:10.1371/journal.pone.0318312)
Supplement: Attachment — Submitted filename: PLOS ONE Reviewer Response 8.30.24.pdf [file pone.0318312.s006.pdf]

Thank you for the opportunity to review this revised manuscript, which details a systematic review protocol on nomenclature and outcomes in children with complex critical illness in the pediatric ICU.

I would like to thank the reviewers for their work in refocusing and reorganizing this manuscript and have the following additional suggestions to continue to clarify and refine the review methods.

<u>Overall</u>

- Integrative vs. systematic review is being used interchangeably throughout manuscript and not all readers have the same understanding on what these two terms mean. I would select one and use throughout.
- There are a lot of similar abbreviations used throughout the manuscript. Preference for a specific term may vary by country, but I would choose pediatric critical care (PCC) OR pediatric intensive care unit (PICU) and use only one to eliminate some of the redundancy in abbreviations.

<u>Abstract</u>

- Review objective is worded differently in the abstract vs. main manuscript and I find the objective in the abstract to be clearer than the one in the main text.

<u>Introduction</u>

- I suggest some reordering of the Introduction for clarity.
    - In the Rationale, discussing CCI in the first paragraph and then PLOS in the second paragraph makes it hard to connect the concepts and understand if they are the same or different ideas. I would move the PLOS paragraph to immediately follow the sentence in the first paragraph which states that children with CCI frequently have PLOS (line 68). Then the Rationale can conclude with the last sentence of the current first paragraph talking about the implications of the lack of clear definition of CCI (lines 68-70).
    - The last paragraph of the Children with Complex Critical Illness section (lines 96-104), which talks about the unique challenges of children with CCI and the UK survey feels out of place in its current location. I think it needs to be moved to earlier in the Introduction, and the intro section should conclude with the paragraph discussing whether a single all-encompassing definition of CCI can be developed (because that is the purpose of your review, lines 87-91).
- The primary outcome is clear to me, but the secondary outcome still feels vague/broad (lines 109-110). I'm not sure what "analyze patient characteristics" means and how it is different from understanding associated criteria used to define CCI stated in the primary outcome. "Assess the outcomes that were investigated", does this mean look at how CCI affected morbidity/mortality?

<u>Materials and Methods</u>

- I do not feel that the inclusion of the concept of a PICO strategy is really needed (line 123) – however if it is kept it needs a reference. Similarly, the PICO Intervention, Comparator, Outcome section (lines 135-138) feels disorganized.
- I am curious why the authors are limiting the definition of CCI they develop to be focused only on the UK, especially if the literature review is international in scope (lines 136-138). If the lack of consensus of CCI is a worldwide problem, shouldn't the solution be acceptable to an international audience? If definition is to be UK-specific, that should be listed as a limitation in the Discussion.
- In the Information Sources section, please move the information about systematic reviews being excluded but references lists reviewed (lines 148-149) to the exclusions section.
- Authors state that all languages are included in the review (line 154) – what about languages the authors are not fluent in?
- The last two sentences of the Search Strategy section are confusing (lines 169-173) – are the authors stating that only articles published after 2014 will be considered? If so, that should be listed earlier in the inclusion/exclusion criteria with this justification.
- The Quality Assessment and Confidence in Cumulative Evidence sections are redundant and should be combined. Some information here is about data synthesis not quality assessment and should be a separate section.
    - What do the authors mean by development of a quality assessment template (lines 195-196). A validated/reliable instrument should be used for quality assessment. If the chosen instrument is the CASP, I don't think the instrument should be modified by the authors?
    - Authors say they will consider conducting a sensitivity analysis for qualitative studies (lines 202-203). This needs defined/referenced.
    - What kind of quantitative data will be pooled statistically (line 210)? The primary focus of the review is understanding how CCI is defined, so this allusion to a meta-analysis is a little confusing. Perhaps it would address the secondary outcome?
- The Outcomes section of the Methods is confusing. Starting at line 223, additional outcomes will be… additional outcomes of the review? Perhaps these are the outcomes you will look at to answer the secondary objective? The process outcomes (line 226-227) are not well-defined and the plans to look at interventions and develop a core outcome set (228-229) is no longer within the scope of the review, correct? The purpose of the review needs to be focused.
- Line 223: Research questions were informed from previous patient engagement in PCC, meaning the authors' previous experience with PPC?

<u>Discussion</u>

- Line 252 – What is PPIE?
