# Supplementary material for: Protocol on a systematic review of nomenclature and outcomes in children with complex critical illness in Paediatric Critical Care: The basis for consensus definition
Source: PLoS One. 2025 Feb 6;20(2):e0318312. doi: 10.1371/journal.pone.0318312 (PMC11801698; doi:10.1371/journal.pone.0318312)
Supplement: Attachment — Submitted filename: Response to Reviewers PLOS ONE letter 1.0.docx [file pone.0318312.s005.docx]

Tai-Heng Chen

Academic Editor

PLOS ONE

Nine Hills Road

Cambridge

United Kingdom

CB2 1GE

31^st^ July 2024

Dear Tai-Heng Chen,

Re: ONE-D-24-25160 Revised Manuscript submission

Protocol on a Systematic review on nomenclature and outcomes in children with complex critical illness in Paediatric Critical Care - The basis for consensus definition.

Thank you for sending valuable comments from the reviewers. We are submitting a revised version of the manuscript that addresses the points raised during the review process.

| Revisions requested | Changes made | Location  (Page number) |
| --- | --- | --- |
| Journal Edits | | |
| 1. Please ensure that your manuscript meets PLOS ONE's style requirements, including those for file naming. | We agree with the reviewers. The style of the manuscript has been updated throughout as per PLOS ONE style templates. |  |
| 2. Please include captions for your Supporting Information files at the end of your manuscript, and update any in-text citations to match accordingly. Please see our Supporting Information guidelines for more information: http://journals.plos.org/plosone/s/supporting-information. | This has now been done with updated in-text citations, thank you. |  |
| Reviewer 1 | | |
| It may be helpful for the authors to include some idea of what makes a "complex critical illness" - I realise that the aim of the overall work is to find a unifying definition of this, but some background on the groups the authors aim to include would be helpful, eg whether they are predominantly referring to children with underlying complex needs who happen to deteriorate and require ICU support vs children previously well children with an acute illness which is complex on the basis of number of systems involved or multiple coexisting pathologies. This is elaborated on in the "eligibilty" section but some more introduction would be useful and would make it clearer to the reader what they are expecting to read about. | Thank you for the input, a section in the introduction has been added to reflect the suggestions. | Page 5 line 76 onwards to 104 |
| In the abstract, the authors say "nationally" - as the journal has international readership, this should be specified "in the UK" | 1. Changed Nationally to conducted in the UK 2. Nationally, changed to ‘in the UK’ | 1. Page 2 line 27 2. Page 3, line 33 |
| The authors plan to limit their search to papers published in the last decade - what is the rationale for this decision? | The search was limited to ten years as the impact of new treatments to increase survival in children has become increasingly common in the past decade with an associated increase in long-stay patients in PICU. As a result, children with complex critical illness are a relatively new concept within PCC. Therefore, the timeline was limited to increase the accuracy and applicability of the review. | Page 3, line 44  Page 9, line 169 |
| Will the search be limited to English language, full-text papers? Will conference abstracts be included? This information should be included in the search strategy. | All languages included – will be translated before data extraction  Conference extracts were excluded but authors were contacted for details of posters and links to manuscripts (if any) | Page 9 line 154  Page 9 line 155 |
| Reviewer 2 | | |
| While this topic is of great importance considering the increasing medical complexity of children admitted to the pediatric ICU, the manuscript is not well organized.  For a better organizational structure, I suggest combining PLOS One requirements for main sections (Introduction, Materials & Methods, Discussion) with the PRISMA-P criteria as subsections within each main section. | Manuscript and abstract order have been changed |  |
| The use of future tense throughout the manuscript is also at times confusing, because it seems that the search strategy has already been formed (Appendix 1) and the trial has already been registered, but these types of activities are stated to occur in the future. Together these two issues make it difficult to clearly understand the review methods. | The search strategy has been formed and the trial registered – this has been amended in the manuscript. |  |
| There should not be references within an abstract. | The references have been removed, thank you. |  |
| If outcomes remain a focus of the review, the core outcomes set developed for pediatric critical care by Fink et al., 2020 should be mentioned. How is your outcomes aim different from this work? Fink EL, Jarvis JM, Maddux AB, et al. Development of a core outcome set for pediatric critical care outcomes research. Contemporary Clinical Trials. 2020;91:105968. doi:10.1016/j.cct.2020.105968 | They no longer remain a focus – we have changed to research priorities on consultation with researchers looking at developing PIC-p research/core outcomes in CCI patients. |  |
| The abstract and main text should follow the same headings. | We thank you for the advice and this now has been amended. |  |
| Only include abbreviations that are used again in the abstract. | AHP has been removed | Page 6 |
| Introduction:  The review purpose/objectives are stated multiple times in multiple different ways. One concise objective or set of aims is needed. | This has been edited to provide a concise aim. | Page 6 line 105 |
| If outcomes remain a focus of the review, the core outcomes set developed for pediatric critical care by Fink et al., 2020 should be mentioned | We thank you for the advice. The development of a core outcomes set is no longer in the remit of this study. |  |
| Additionally, the review objective(s) seems quite broad. Understanding how pediatric chronic critical illness (PCCI) is defined, who the children with PCCI are, and the outcomes they are experiencing feels like 3 separate literature reviews, and will be difficult to complete systematically using one search strategy, data extraction tool, etc. | Investigating how children with complex critical illness are currently defined and what is the criteria of each definition in the literature will allow for the next steps of consensus definition development.  The outcomes from each study will be analysed to form a knowledge base of current topics of interest in this patient population. |  |
| A clearer reason is needed for how this review is different from the Zorko et al., 2023 scoping review.  Edwards et al., 2022 review also needs mentioned earlier, and how it is distinct from the review you are undertaking. | Edwards et al. conducted a concise review in 2022(2), this review plans to expand on the topic in depth and breadth to provide an up-to-date analysis of nomenclature, criteria, and outcomes of interest of children with CCI for the basis of consensus definition development. Following this, Zorko et al conducted a scoping review in January 2023 which looked specifically at the term ‘chronic critical illness’ within PICU(6). The review included studies which provided a specific case definition for chronic critical illness or prolonged stay. As such, Zorko’s review differs as it did not encompass medical complexity at admission, repeated admissions or technological dependence. It also does not provide an overview of all current nomenclature and associated criteria for children with complex critical illness | Page 6 line 87 |
| I do not believe Table 1 is needed, and in fact is confusing as the authors have already stated that there are not commonly accepted definitions for these concepts. I think it would be adequate to state that chosen search terms were based on concepts identified in the Edwards et al., 2022 review. | Table has been removed and ‘The chosen search terms were based on concepts identified in the Edwards et al., 2022 review.’ Has been added |  |
| Things being mentioned in the Introduction belong in the Methods (e.g., eligibility criteria, types of sources). | They have now been removed – thank you. |  |
| Article keywords do not belong in the main text. | They have now been removed, thank you. |  |
| What is the COMET theoretical framework? It is mentioned without reference or explanation. Is this where the following Concept and Context sections come from, because I also found those a bit confusing and redundant from what had already been described. | They have now been removed – thank you. |  |
| Again, exploring all types of literature with such a broad research question feels quite difficult. Is there a specific reason grey literature needs to be included? | Thank you for this consideration, we have limited the grey literature to “Tier 1” only. Including grey literature will allow knowledge and evidence from the ‘clinical floor’ of PCC to be considered. | Page 9 151 to 154 |
| Won’t the inclusion of systematic reviews be redundant given the inclusion of original research articles? | We agree with the reviewer and only relevant citations will be reviewed as per the next point. | Page 149 to 151 |
| Perhaps the reference list of reviews can be searched for relevant citations but don’t need to be included themselves? | We agree with the reviewer and the reference list of reviews will be searched for relevant citations. | Page 149 to 151 |
| Methods: Authors should state that they did (not “will”) follow PRISMA-P when reporting protocol and reference Appendix 3 at the beginning of methods section. | Has now been changed to reflect this. | Page 5 line 104-106 |
| Use PRISMA-P criteria to organize manuscript. | We agree with the reviewer, and this has been done. |  |
| Use of multiple article reviewers and close consultation with librarian are strengths of the review methods | Thank you |  |
| Authors state that they “will” register protocol on PROSPERO, but it seems that they already have? Please reference this in Abstract and Methods. | This has been changed. | Page 7 line 118 |
| Appendices are incorrectly numbered/ identified in text | We thank the reviewer, and this has now been changed and updated. |  |
| A pilot test is mentioned in Study/Source of Evidence selection section, what does this mean? | This has been removed. I tested that the software was functioning correctly, accessible by the whole team, and the criteria were correctly inserted. But this does not have to be part of the manuscript as it is standard procedure, so it has been removed – thank you for the suggestion. | Page 6 line 161 |
| CASP abbreviation should be defined and referenced in Quality Assessment section. A description of the tool is needed. | This has been done – thank you for the suggestion. | Page 7 Line 180 |
| Patient and public involvement section feels vague, is there a specific parent advisory group that was consulted on methods? | A parent was included in this review first. Following the systematic review a parent advisory group is being formed for the consensus work following the review. |  |
| Dissemination strategies are mentioned multiple times and feel redundant (speaks to need for better manuscript organization). Authors state they “will” publish protocol in academic pediatric critical care literature which feels confusing to read in a protocol paper. Also, while PLOS ONE is a great journal, I would not call it a pediatric critical care journal? | This has been moved to the discussion section and language edited. | Page 13 line 252 |
| Multiple ethics sections – again feels redundant. | Amalgamated to one section. | Page 13 Line 236 |
| Discussion/Conclusion • Discussion section should be a discussion of the review protocol, not a report of what will be discussed in the results manuscript. Should include strengths/ limitations of review methods, how it builds on previous reviews, and how this review will be used to build the consensus definitions (which is currently included in the Methods and should be moved) | We thank you for your valuable advice and this has been changed to reflect your comments. | Page 13 and 14 line 246-258 |

We thank both reviewers for taking the time to review this manuscript and providing valuable feedback and reflections.

Yours sincerely

Sofia Cuevas-Asturias

BSc MBChB MRCPCH

MD(Res) Clinical Research Fellow at Imperial College London

On behalf of the Author group:

Hannah K Mitchell, Claire Rafferty, Will Tremlett, Padmanabhan Ramnarayan, Natalie Pattison
