# Supplementary material for: Protocol on a systematic review of nomenclature and outcomes in children with complex critical illness in Paediatric Critical Care: The basis for consensus definition
Source: PLoS One. 2025 Feb 6;20(2):e0318312. doi: 10.1371/journal.pone.0318312 (PMC11801698; doi:10.1371/journal.pone.0318312)
Supplement: Attachment — Submitted filename: Response to Reviewers PLOS ONE letter 13.12.24.docx [file pone.0318312.s007.docx]

Tai-Heng Chen

Academic Editor

PLOS ONE

Nine Hills Road

Cambridge

United Kingdom

CB2 1GE

13^th^ December 2024

Dear Tai-Heng Chen,

Re: PONE-D-24-25160R1 Revised Manuscript submission

Protocol on a Systematic review on nomenclature and outcomes in children with complex critical illness in Paediatric Critical Care - The basis for consensus definition.

Thank you for sending valuable comments from the reviewers. We are submitting a re-revised version of the manuscript that addresses the points raised during the review process.

| Revisions requested | Changes made | Location  (Page number) |
| --- | --- | --- |
| Journal Edits | | |
| Integrative vs. systematic review is being used interchangeably throughout manuscript and not all readers have the same understanding on what these two terms mean. I would select one and use throughout. | I have selected systematic, thankyou | Various throughout the document. |
| There are a lot of similar abbreviations used throughout the manuscript. Preference for a specific term may vary by country, but I would choose pediatric critical care (PCC) OR pediatric intensive care unit (PICU) and use only one to eliminate some of the redundancy in abbreviations. | I have changed the term to Paediatric Critical Care (PCC). | Various throughout the document. |
| Review objective is worded differently in the abstract vs. main manuscript and I find the objective in the abstract to be clearer than the one in the main text | I have matched the objectives in the abstract and main manuscript – thank you for this. | Line 115 |
| Reviewer 1 | | |
| I suggest some reordering of the Introduction for clarity:  In the Rationale, discussing CCI in the first paragraph and then PLOS in the second paragraph makes it hard to connect the concepts and understand if they are the same or different ideas. I would move the PLOS paragraph to immediately follow the sentence in the first paragraph which states that children with CCI frequently have PLOS (line 68). Then the Rationale can conclude with the last sentence of the current first paragraph talking about the implications of the lack of clear definition of CCI (lines 68-70). | Thank you for the suggestion to increase clarity, I have followed the advice. | Line 68-75 |
| The last paragraph of the Children with Complex Critical Illness section (lines 96- 104), which talks about the unique challenges of children with CCI and the UK survey feels out of place in its current location. I think it needs to be moved to earlier in the Introduction, and the intro section should conclude with the paragraph discussing whether a single all-encompassing definition of CCI can be developed (because that is the purpose of your review, lines 87-91) | Thank you for your suggestion, the survey has been moved to earlier. | Lines 88-91 |
| The primary outcome is clear to me, but the secondary outcome still feels vague/broad (lines 109-110). I’m not sure what “analyze patient characteristics” means and how it is different from understanding associated criteria used to define CCI stated in the primary outcome. “Assess the outcomes that were investigated”, does this mean look at how CCI affected morbidity/mortality? | We would like to assess what aspects of care/outcomes are repeatedly investigated within this patient population e.g. mortality, morbidity, length of stay, risk of re-admission etc. By looking at the outcomes assessed, we can suggest what are the current outcomes of priority to aid the future development of research priorities within CCI. |  |
| I do not feel that the inclusion of the concept of a PICO strategy is really needed (line 123) – however if it is kept it needs a reference. Similarly, the PICO Intervention, Comparator, Outcome section (lines 135-138) feels disorganized. | Line 123 has been re-worded to: The following criteria will be considered for this review and the PICO section has been re-worded. Thank you for highlighting this. | Line 137-155 |
| I am curious why the authors are limiting the definition of CCI they develop to be focused only on the UK, especially if the literature review is international in scope (lines 136-138). If the lack of consensus of CCI is a worldwide problem, shouldn’t the solution be acceptable to an international audience? If definition is to be UK-specific, that should be listed as a limitation in the Discussion. | Thank you for your thoughts. I have discussed this further in the limitations section as suggested. | Line 273-275 |
| In the Information Sources section, please move the information about systematic reviews being excluded but references lists reviewed (lines 148-149) to the exclusions section. | This has been done – thank you for your comments. | Line 156 |
| Authors state that all languages are included in the review (line 154) – what about languages the authors are not fluent in? | Authors are fluent in English, Spanish, and Portuguese and will use online translation tools for other languages. | Line 154 |
| The last two sentences of the Search Strategy section are confusing (lines 169-173) – are the authors stating that only articles published after 2014 will be considered? If so, that should be listed earlier in the inclusion/exclusion criteria with this justification. | Thank you for your input, I have moved the statement to the exclusion criteria and included reasoning. | Line 175-176 |
| The Quality Assessment and Confidence in Cumulative Evidence sections are redundant and should be combined.  What do the authors mean by development of a quality assessment template (lines 195-196). A validated/reliable instrument should be used for quality assessment. If the chosen instrument is the CASP, I don’t think the instrument should be modified by the authors? | Thank you for your input, the sections have been put together. The CASP checklists are to be used without modification of the questions. This has been amended. | Line 222-242 |
| Authors say they will consider conducting a sensitivity analysis for qualitative studies (lines 202-203). This needs to be defined/referenced. | Thank you for your insight, this has been modified and qualitative studies will be evaluated using Grade-CERQual. | Line 230-237 |
| What kind of quantitative data will be pooled statistically (line 210)? The primary focus of the review is understanding how CCI is defined, so this allusion to a meta-analysis is a little confusing. Perhaps it would address the secondary outcome? | If there was enough homogeneity between definitions, we wanted to consider pooling outcome data for a meaningful data analysis. But this is unlikely to be the case so it has been removed. Thank you for the input. | Line 239-243 |
| Some information here is about data synthesis not quality assessment and should be a separate section. | This has been removed and placed into the correct section. Thank you. |  |
| The Outcomes section of the Methods is confusing. Starting at line 223, additional outcomes will be… additional outcomes of the review? Perhaps these are the outcomes you will look at to answer the secondary objective? The process outcomes (line 226-227) are not well-defined and the plans to look at interventions and develop a core outcome set (228- 229) is no longer within the scope of the review, correct? The purpose of the review needs to be focused | Thank you for your comments. All the points made have been addressed and modified. | Line 243-254 |
| Line 223: Research questions were informed from previous patient engagement in PCC, meaning the authors’ previous experience with PPC? | I have clarified the language to:  The research questions have been developed as a result of the author teams' engagement with children with complex critical illness and their families in PCC. | Line 235 |
| Discussion section: Line 252 – What is PPIE? | Patient and public involvement and engagement. This has now been clarified, thank you. | Line 254 |

We thank both reviewers for taking the time to review this manuscript and providing valuable feedback and reflections.

Yours sincerely,

Sofia Cuevas-Asturias

BSc MBChB MRCPCH

MD(Res) Clinical Research Fellow at Imperial College London

On behalf of the Author group:

Hannah K Mitchell, Claire Rafferty, Will Tremlett, Padmanabhan Ramnarayan, Natalie Pattison
